# Facile Interfacial Synthesis of Densely Spiky Gold Nano-Chestnuts With Full Spectral Absorption for Photothermal Therapy

**DOI:** 10.3389/fbioe.2020.599040

**Published:** 2020-10-26

**Authors:** Zhiping Wan, Jinmao Gu, Yining Wang, Jun Qian, Junle Zhu, Feng Chen, Haoheng Wang, Huairui Chen, Chun Luo

**Affiliations:** ^1^Department of Neurosurgery, Tongji Hospital, Tongji University School of Medicine, Shanghai, China; ^2^Center of Reproductive Medicine, Shanghai Changzheng Hospital, Shanghai, China

**Keywords:** SGNCs, photothermal effect, malignant tumor, spike (S), spectral absorption

## Abstract

The gold nanostructure is regarded as the most promising photothermal agent due to its strong localized surface plasma resonance (LSPR) effect. In particular, the gold nanostructures with sharp spikes on the surface have higher optical signal enhancement, owing to the sharp tips drastically enhancing the intense nanoantenna effect. However, current approaches for the synthesis of spiky gold nanostructures are either costly, complicated, or uncontrollable. Herein, we report a novel strategy to synthesize gold nano-chestnuts (SGNCs) with sharp spikes as an excellent photothermal agent. The SGNCs were prepared by a facile one-pot interfacial synthetic method, and their controllable preparation mechanism was acquired. The SGNCs exhibited ideal full-spectrum absorption and showed excellent photothermal effect. They have a photothermal conversion efficiency (η) as high as 52.9%, which is much higher than traditional photothermal agents. The *in vitro* and *in vivo* results show that the SGNCs could efficiently ablate the tumor cells. Thus, the SGNCs have great potential in photothermal therapy applied in malignant tumors.

## Introduction

Tumors, especially malignant tumors, have become one of the greatest threats to human health. Many strategies were devised for oncotherapy, and photothermal therapy (PTT) is the newest and most promising strategy among them, which uses PTT agents to convert the near-infrared (NIR) energy to heat for hyperthermia of tumors (Huang et al., 2017; Liu et al., 2019; Yang et al., 2019; Fernandes et al., 2020; Wang Y. et al., 2020; Zhang et al., 2020). Lots of PTT agents have been reported, such as semiconductors (transfer metal sulfide and transfer metal oxide), carbon materials (carbon nanotube), and noble metals (Liu et al., 2014, 2019; Huang et al., 2017). Among them, gold nanostructures (GNSs) are the most promising PTT agent, which could obtain strong localized surface plasma resonance (LSPR) due to their morphology and size (Cao et al., 2014; Furube and Hashimoto, 2017; Kong et al., 2020; Tabish et al., 2020). GNSs have also been proven not only as an efficient imaging agent for tumor diagnosis but also as a theranostic agent for thermal ablation of tumor cells, due to their excellent photothermal effect and tunable optical properties (Xia et al., 2011; Ren et al., 2015; An et al., 2017; D’Acunto, 2018; Norouzi et al., 2018). For *in vivo* applications, the LSPR adsorption of GNSs must be optimized to the NIR region where light has high tissue transparency and penetration depth. To date, a variety of NIR-absorbing GNSs, such as nanorods (Xu et al., 2020), nanoshells (Xuan et al., 2016), nanocones (Zhang et al., 2013), nanocups (He et al., 2012), nanocages (Xia et al., 2011), and nanostars (Zhang et al., 2019), have been synthesized and widely explored for biomedical sensing, imaging, and therapy. As viable alternatives, GNSs with sharp spikes on the surface have emerged and attracted the most attention for a higher optical signal enhancement, owing to their sharp tips drastically enhancing an intense nanoantenna effect (de Silva Indrasekara et al., 2018). This feature leads to a high LSPR effect and strong adsorption for PTT. Currently, there are some reports on the synthesis of such spiky gold nanostructures (SGNSs), for example, peptide-modulated (Yuan et al., 2012; Yan et al., 2018) or seed-mediated (Sanchez-Gaytan and Park, 2010; de Silva Indrasekara et al., 2018) growth of SGNSs. However, these approaches are either tedious, complicated, or uncontrollable with low quality of spikes. Moreover, SGNSs synthesized by these methods do not exhibit full spectrum absorbance, although they are highly desired for photo-based therapy of ablating tumors.

**FIGURE 1:**
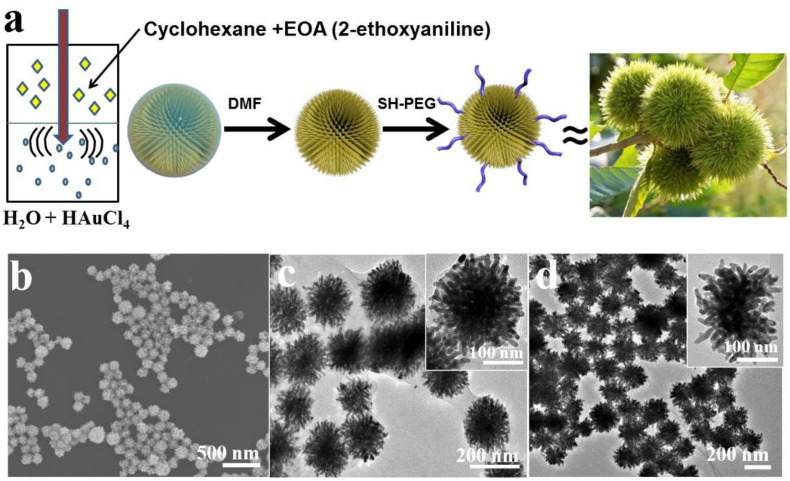
**(a)** Schematic illustration for the synthesis of SGNCs. **(b)** SEM images of prepared SGNCs from EOA. **(c,d)** TEM images of SGNCs before and after washed by DMF.

In the current study, we report a novel, facile, scalable strategy for one-pot synthesis of uniform chestnut-like GNSs densely populated with sharp spikes on the surface (SGNCs). The SGNCs were obtained through facile reduction of a gold precursor (an aqueous solution of HAuCl_4_) by 2-ethoxyaniline (EOA) at an oil/water interface to modulate the highly branched growth of GNSs (Figure 1a). The prepared SGNCs show strong absorption, from the UV to the NIR region (400–1000 nm) instead of mono- or bimodal absorption for traditional gold nanoparticles (GNPs), which is due to the dense surface coverage of spikes with varying diameters and lengths. Furthermore, the SGNCs exhibited excellent PTT effect, *in vitro* and *in vivo*, which can efficiently ablate tumors under the irradiation of an 808-nm laser. Thus, SGNCs have a great potential as an excellent PTT agent for tumor treatment.

## Experiment

### Materials

2-Ethoxyaniline, gold (III) chloride hydrate (HAuCl_4_, 99.99%), and cyclohexane were purchased from Sigma-Aldrich and used as received. Dulbecco’s modified Eagle’s medium (DMEM), 2-(4-Amidinophenyl)-6-indolecarbamidine dihydrochloride (DAPI), and fluoresceinamine isomer I were obtained from Sigma-Aldrich. Fetal bovine serum (FBS), dimethyl sulfoxide (DMSO), trypsin-EDTA, and penicillin/streptomycin (5000 U ml) were purchased from Thermo Fisher Scientific.

### Characterization

The SGNCs were imaged using a scanning electron microscope (SEM, S-4800) and a transmission electron microscope (TEM, JEM-2100F). The images of thermal results were recorded by an SC300 infrared camera (FLIR, VA, United States) and analyzed with IR images software (FL-IR).

### Synthesis of SGNCs

In a typical synthesis, 2.04 mg of HAuCl_4_ (6 μmol) was slowly dissolved in 5 ml of deionized water. Subsequently, 0.5 ml of an EOA cyclohexane solution (30 mM) was added dropwise to the above aqueous solution. The mixture was then treated with ultrasound. The SGNCs were collected by centrifugation and washed three times with deionized water. Then, the SGNCs were dispersed in DMF overnight. The SGNCs were further washed five times. Then, SH-PEG was mixed with the SGNCs overnight and then the PEG-modified SGNCs were collected by centrifugation.

### Synthesis of GNPs

To prepare GNPs, 1 ml of a 20 mg/ml HAuCl_4_ solution was injected into 500 ml of boiling water containing citrate under stirring, and then 3 ml of 10 mg/ml sodium citrate was added into the above solution. The temperature of solution was decreased to 85°C, after refluxing for 40 min. Furthermore, 1 ml of 20 mg/ml HAuCl_4_ solution and 3 ml of 10 mg/ml sodium citrate solution was added to the above solution. Finally, GNPs were obtained by centrifugation and washing.

### Photothermal Performances *in vitro*

The solutions of SGNCs and GNPs at a concentration of 300 μg/ml were irradiated with an 808-nm laser (1.0 W/cm^2^). The temperature of the solutions was recorded by an infrared camera (FLIR, VA, United States). To estimate the cell mortality rate under laser irradiation, the MTT assay was used to evaluate it. U87MG cells were seeded in 96-well plates, and then these cells were incubated overnight at 37°C. Further, cells were rinsed with PBS solution (pH 7.4); the cells were incubated under different concentrations of SGNCs and GNPs for 4 h at 37°C under the same conditions. Then, PBS was used to rinse cells a second time and injected in 200 μl of new culture medium after being irradiated using an 808-nm laser with ∼1 W/cm^2^ for 5 min. After the irradiation, cells were incubated for another 24 h in an incubator at 37°C. Procedures for the control group were identical to the experimental group except for the irradiation. To further identify the cell viability, Calcein AM and ethidium homodimer-1 were used to stain the cells to distinguish live (showing green) and dead (showing red) cells.

### *In vivo* Photothermal Ablation and Therapy for Tumors

The mice for *in vivo* experiments were conducted following the protocols of the Shanghai Committee for the Accreditation of Laboratory Animal. All processes have been approved by the Shanghai Science and Technology Commission (Laboratory Animal Use Permit Number: SYXK2019-0005; Shanghai, China). All operations were performed after sodium pentobarbital anesthesia with all efforts made to minimize the suffering of mice.

When the tumor reached a size of ∼6 mm, the U87MG tumor-bearing mice were randomly divided into three groups (four mice per group): group I: Control, group II: GNPs, and group III: SGNCs. For group II and group III, GNPs and SGNCs with a dose of 5 mg/kg were i.t., injected. Mice in the control group were did not receive treatment. Mice in groups II and III were then irradiated by an 808-nm laser at a power density of 1 W/cm^2^ for 8 min, and an infrared camera was applied to monitor the temperature changes. The mouse weights and tumor sizes were measured after treatment every 2 days for 14 days. At day 14, mice were all killed, and the major organs were isolated for slides. The slides were stained with hematoxylin/eosin and then observed with a microscope.

## Results and Discussion

The SGNCs were obtained by an aqueous solution of Au precursor (HAuCl_4_) mixed with a solution of EOA in cyclohexane under moderate sonication. The redox reaction between Au^3+^ and EOA led to the concurrent polymerization of EOA into spherical poly(EOA) NPs and the formation of uniform SGNCs on the surface of poly(EOA) (Figures 1b–d). Over time, the resulting SGNCs detached from the poly(EOA) NPs, leaving a thin layer of poly(EOA) on the SGNC surface (Figure 1c). The poly(EOA) layer can be washed away using dimethylformamide (DMF), which is a good solvent for poly(EOA) (Figure 1d). The surface of the SGNCs is densely populated with sharp spikes with average widths and lengths of ∼12 nm and ∼40 nm, respectively (Figures 1b,c). The average particle size was measured to be ∼190 nm (Supplementary Figure 1). The surface of the SGNCs was subsequently modified with polyethylene glycol (PEG) to improve their biocompatibility in biological applications.

**FIGURE 2:**
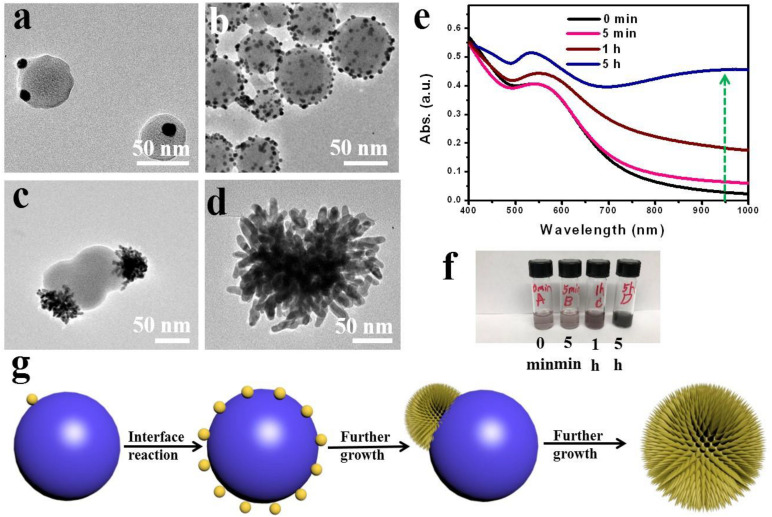
Time-dependent growth of SGNCs. **(a–d)** TEM images of SGNCs obtained in the same reaction proceeded for 0 min **(a)**, 15 min **(b)**, 60 min **(c)**, and 5 h **(d)**, respectively. **(e,f)** The UV–vis spectra and digital picture of SGNCs in water at various reaction times: 0, 5, 60 min, and 5 h. **(g)** The mechanism of preparing SGNCs.

To understand the growth mechanism of spikes, we evaluated the time-dependent formation of SGNCs using TEM imaging and UV–vis spectroscopic analysis. As shown in Figures 2a–d, immediately after mixing the reactant solutions, bits of tiny spherical GNPs (∼10 nm in diameter) were rapidly formed on the surface of poly(EOA) NPs (∼60 nm in diameter). Approximately 5 min after mixing, many small GNPs presented on the surface of the polymer spheres, and the diameter of polymer spheres increased from 50 to 70 nm, suggesting the further reduction of Au^3+^ to Au^0^ by EOA. The Au deposition continued, spiky gold thorn gradually grew on the poly(EOA) particles, while the smaller GNPs disappeared after 5 h. We presume that a ripening process occurred, leading to the dissolution of the small GNPs and re-deposition on a few seed GNPs immobilized on the polymer particles. The reaction was completed in ∼5 h to form a final product of SGNCs of ∼190 nm. UV–vis spectra at the corresponding time points show a gradual increase in the NIR absorption of the nanostructures as the reaction proceeded (Figures 2e,f). At the first stage of preparation of SGNCs, there are bits of GNPs in the poly(EOA) NPs; the absorption peak of ∼530 nm is due to the longitudinal LSPR properties of GNPs. With the growth of the GNPs, the LSPR peaks shifted to red and became broader due to plasmonic coupling between particles (Hu et al., 2006). Finally, when the SGNCs were prepared, the obvious longitudinal peak vanished and formed a broad bandwidth, which could be attributed to the increasing number of branches and lengths of GNSs (Cheng et al., 2012; de Silva Indrasekara et al., 2018). Thus, we deduce the preparation mechanism of SGNCs as in Figure 2g.

**FIGURE 3:**
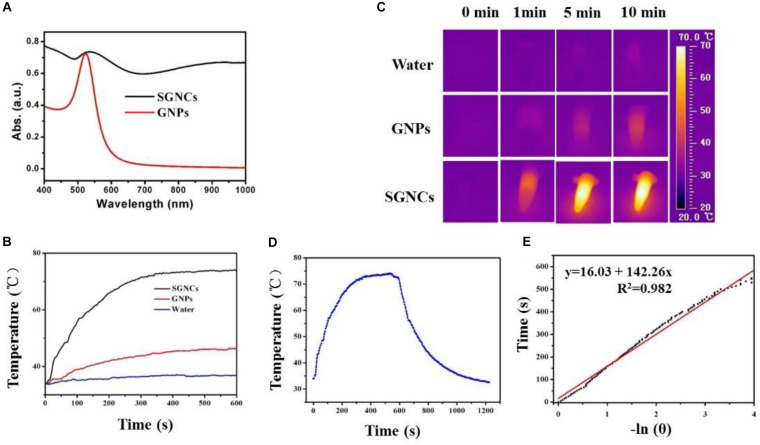
**(A)** UV–vis-NIR absorbance spectra of GNPs and SGNCs. The temperature elevation **(B)** and thermal images **(C)** of water, GNPs, and SGNCs at a concentration of 300 μg/ml with time under the irradiation of an 808-nm laser with a power density of 1 W/cm^2^. **(D)** The temperature change with irradiation for 10 min and nature cooling process. **(E)** Graph for negative natural logarithm versus cooling process to calculate the photothermal conversion efficiency (η).

We further assessed the potential use of SGNCs as theranostic agents in PTT and thermal imaging. As shown in Figure 3A, SGNCs exhibited a significantly higher absorption in the NIR window (e.g., 808 nm) when compared with GNPs at the same concentration of Au element. To further investigate the photothermal performances of the SGNCs and GNPs, the dispersions of SGNCs and GNPs at a concentration of 300 μg/ml and pure water were irradiated under an 808-nm laser with an energy density of 1 W/cm^2^. The temperature of the pure water had an insignificant rise, and a small increase (less than 10°C) in temperature was observed for the GNP dispersion, while SGNC dispersion increased to 73.8°C within 600 s (Figure 3B). Owing to the enhanced LSPR adsorption, the temperature of SGNCs exhibited an approximately three-fold increase compared to that of GNPs. The higher photothermal effect of SGNCs than GNPs was visually observed by thermal images (Figure 3C). The photothermal conversion efficiency of the SGNCs was determined according to the previous method (Tian et al., 2011; Liu et al., 2014): $\eta =\frac{h\,s\,\left(T_{\text{max}}-T_{\text{surr}}\right)-Q_{\text{Dis}}}{I\,\left(1-10^{-A\,808}\right)}$.

After the temperature of the dispersion reached a steady state, the laser was turned off and the temperature variation was recorded (Figure 3D). The maximum steady temperature (*T*_max_) minus environmental temperature (*T*_Surr_) of the solution of the SGNCs was 41.5°C. The slope of the linear fitting curve acquired from the cooling process was used to calculate the τ_s_ (Figure 3E). The equation is *t* = -τ_s_ln[(*T* - *T*_Surr_)/(*T*_max_ - *T*_Surr_)]. Then, the equation of *hs* = (*m*_D_*C*_D_)/τ_s_ was used to calculate the *hs*. Finally, the 808-nm laser heat conversion efficiency (η) of the SGNCs is calculated to be 52.9%, which is much higher than traditional photothermal agents, such as gold stars (40.1%) (Yu et al., 2020), gold nanorods (23.7%) (Tian et al., 2011), CuS (38.0%) (Liu et al., 2014), and Bi nanodots (30%) (Lei et al., 2017). Recent photothermal agents, such as FeCo@C-PEG nanoparticles (Song et al., 2020), Nano(O-Nonacene)-PEG (Yin et al., 2019), and upconverting covalent organic framework (COF) (Wang P. et al., 2020), are all ingeniously designed for PTT. However, the presented densely spiky gold nano-chestnuts are simply designed and showed excellent PTT effect for tumors. The SGNCs exhibit higher photothermal conversion efficiency (η) than other photothermal agents, which could be attributed to the strong spectral adsorption bandwidth in the NIR region. The SGNCs with dense SGNSs enhance the electromagnetic field for the tips of the branched particles, which leads to strong LSPR adsorption (Cheng et al., 2012; de Silva Indrasekara et al., 2018). These results showed that the SGNCs can be used as an excellent PTT agent.

**FIGURE 4:**
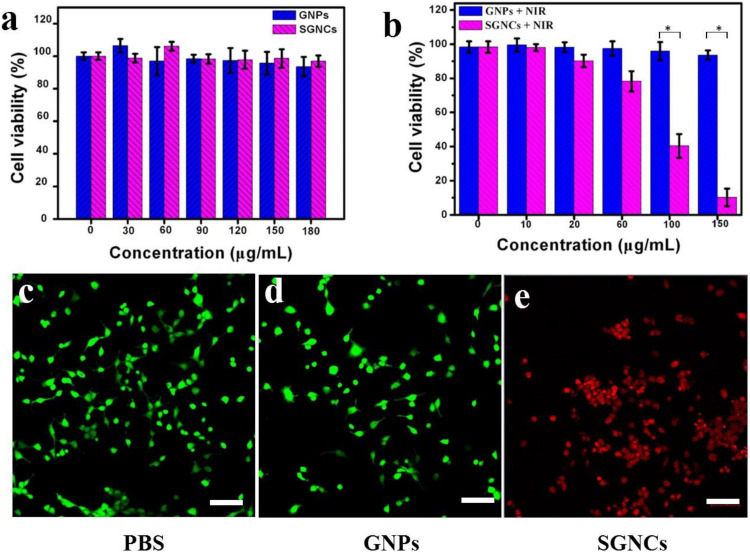
In vitro cytotoxicity of GNPs and SGNCs to U87MG cells after incubation for 12 h **(a)** with no laser and **(b)** with laser irradiation. **(c)** Laser only and **(d,e)** incubation with GNPs and SGNCs and then irradiation with an 808-nm laser (1 W/cm^2^ for 5 min) at a concentration of 150 μg/ml. Scale bar: 100 μm.

The cytotoxicity of SGNCs and their performance in PTT of tumor cells were evaluated in vitro using U87MG cells. As illustrated in Figure 4a, the cell viability remained relatively high after incubation with GNPs or SGNCs with a concentration as high as 180 μg/ml, indicating good biocompatibility. The viability of cells incubated with Au nanostructures and exposed to an 808-nm laser was further investigated using the MTT assay (Figure 4b). The cell viability of the GNP-treated groups showed no obvious divergence from control groups even at concentrations of ∼150 μg/ml. In contrast, the SGNC-treated group showed a significant reduction in cell viability with increasing SGNC concentration upon laser irritation. The PTT of tumor cells was further confirmed by staining living cells of the aforementioned groups (Figures 4c–e). Most of the cells were killed by the SGNC-treated group due to the excellent photothermal effect. Nevertheless, tumor cells’ death was not significant in the GNP-treated groups and the control group. GNPs serve as robust PTT agents toward tumor therapy, which might trigger cancer cell death by a programmed apoptosis with the activation of the caspase-3 pathway (Ali et al., 2016; Wang et al., 2018).

**FIGURE 5:**
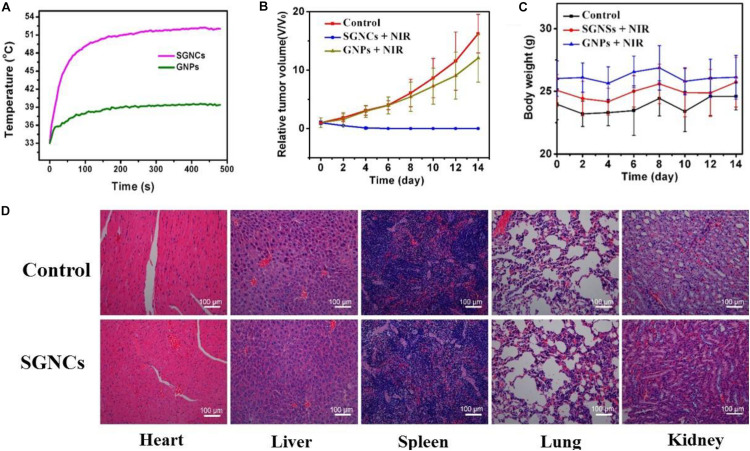
**(A)** The U87MG tumor-bearing mice of the GNP and SGNC group were irradiated with NIR laser for 8 min. The volume change of tumors **(B)** as well as the body weight **(C)** of the tumor-bearing mice were measured every 2 days in different groups. **(D)** H&E-stained pictures of organs (heart, liver, spleen, lung, and kidney) of control group and SGNC group mice, showing no organ damage evidence contrast with the control group and the SGNC group.

The mice were separated into three groups casually (four mice for each group) to elevate the PTT effect. The mice in the control group received no treatment. The mice in the GNP and SGNC groups were intravenously injected with 100 μl of 5 mg/kg GNPs and 100 μl of 5 mg/kg SGNCs, respectively. Then, the GNP and SGNC groups were irradiated with NIR laser for 8 min at the tumor site. For the SGNC group, the tumor temperature rapidly reached 50°C, which is high enough to irreversibly kill the tumor cells. While the tumor temperature of the GNP group only increased to below 45°C (Figure 5A), which is not enough to kill tumor cells (Liu et al., 2020). After NIR irradiation, the volume change of tumors along with the body weight of the tumor-bearing mice were measured every 2 days. We found that the SGNC group obviously inhibited the growth of tumor cells, even completely ablating the tumor (Supplementary Figure 2), while tumor growth had no obvious difference between the GNP group and the control group (Figure 5B). Moreover, mice in the SGNC and GNP groups had no significant weight loss during treatment, implying negligible systemic toxicity of nanomaterials (Figure 5C). Furthermore, the pictures of organs’ H&E stain show that tissue structure and cells had no obvious difference between the SGNC and the PBS group (Figure 5D), further proving the safety of SGNC treatment.

## Conclusion

In summary, we have developed a novel facile approach for the preparation of SGNCs with high density of spikes and full-spectrum absorption and explored its controllable preparation mechanism. The SGNCs had a photothermal conversion efficiency as high as 52.9%. Moreover, the SGNCs could completely ablate the tumors under NIR irradiation of the 808-nm laser, showing better performance than GNPs due to the high density of spike structure. Therefore, SGNCs can serve as good PT agents for tumor therapy.

## Data Availability Statement

The original contributions presented in the study are included in the article/Supplementary Material, further inquiries can be directed to the corresponding author/s.

## Ethics Statement

The animal study was reviewed and approved by Shanghai Committee for the Accreditation of Laboratory Animal; Shanghai Science and Technology Commission (The Laboratory Animal Use Permit Number: SYXK2019-0005’ Shanghai, China).

## Author Contributions

ZW: data curation, and writing – original draft preparation. JG: conceptualization and investigation. YW: visualization. JQ: methodology. JZ: software. FC: investigation. HW: writing – reviewing. HC: supervision. CL: supervision and editing. All authors reviewed the manuscript.

## Conflict of Interest

The authors declare that the research was conducted in the absence of any commercial or financial relationships that could be construed as a potential conflict of interest.
