# Supplementary material for: Facile Interfacial Synthesis of Densely Spiky Gold Nano-Chestnuts With Full Spectral Absorption for Photothermal Therapy
Source: Front Bioeng Biotechnol. 2020 Oct 26;8:599040. doi: 10.3389/fbioe.2020.599040 (PMC7649415; doi:10.3389/fbioe.2020.599040)
Supplement: Supplementary file 1 [file Data_Sheet_1.docx]

Supporting Information

**Facile interfacial synthesis of densely spiky gold nano-chestnuts with full spectral absorption for photothermal therapy**

Zhiping Wan,‡^a^, Jinmao Gu,‡^a^ Yining Wang,‡^b^ Jun Qian,^a^ Junle Zhu,^a^ Feng Chen,^a^ Haoheng Wang,^a^ Huairui Chen,^a^* Chun Luo^a^*

^a^ Department of Neurosurgery, Tongji Hospital, Tongji University School of Medicine, Shanghai, China

^b^ Center of Reproductive Medicine, Shanghai Changzheng Hospital, Shanghai, China

^‡^These authors contributed equally in this work.

* Corresponding author

Chun Luo(Email: boyluochun@126.com);

Huairui Chen(Email:chen13761626536@163.com)

**Supplementary Figures**


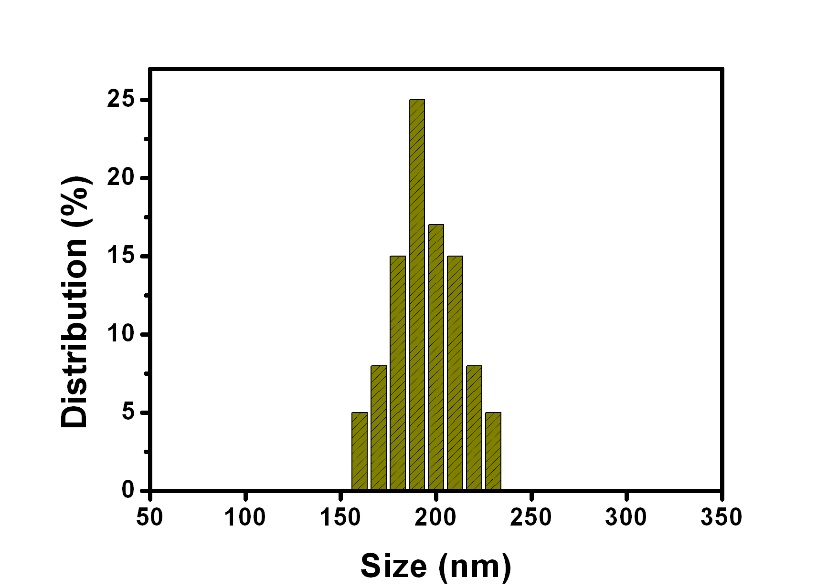


**Fig. S1**. The size distribution of SGNCs.


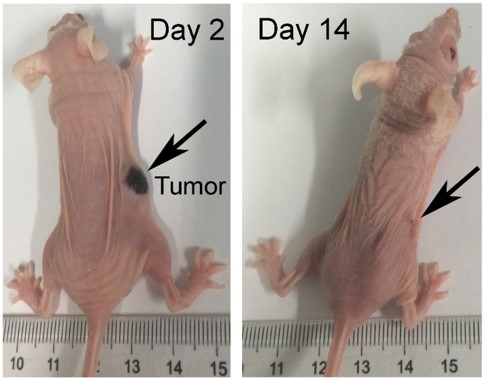


**Fig. S2.** The photographs of the typical mouse by treatment of SGNCs + NIR at day 2 and day 14.
